# Patient-reported quality of life and pain after permissive weight bearing in surgically treated trauma patients with tibial plateau fractures: a retrospective cohort study

**DOI:** 10.1007/s00402-018-3088-5

**Published:** 2018-12-06

**Authors:** Pishtiwan Hassan Shaker Kalmet, Yvette Y. Van Horn, Sebastian Sanduleanu, Henk A. M. Seelen, Peter R. G. Brink, Martijn Poeze

**Affiliations:** 1 Department of Traumatology, Trauma Surgery, Maastricht University Medical Center, P. Debyelaan 25, 6229 HX Maastricht, The Netherlands; 2 Department of Amputation, Traumatology and Orthopaedics, Adelante Rehabilitation Center, Hoensbroek, The Netherlands; 3 Adelante Center of Expertise in Rehabilitation and Audiology, Hoensbroek, The Netherlands; 4 Research School CAPHRI, Department of Rehabilitation Medicine, Maastricht University, Maastricht, The Netherlands; 5 Nutrim School for Nutrition, Toxicology and Metabolism, Maastricht University, Maastricht, The Netherlands

**Keywords:** Tibial plateau fractures, Trauma patients, Complications, Rehabilitation, Weight bearing, Quality of life

## Abstract

**Introduction:**

A Dutch survey among orthopedic surgeons and trauma surgeons showed that almost 90% of the surgeons do not follow protocols regarding the weight bearing aftercare for tibial plateau fractures. Clinical studies comparing permissive weight bearing (PWB) versus restricted weight bearing (RWB) after surgically treated tibial plateau fractures are not available. The aim of this study was to inventory potential differences in quality of life and pain, and number of complications in patients with surgically treated tibial plateau fractures who followed a PWB regime, relative to those that followed a RWB regime.

**Materials and methods:**

This retrospective cohort study included surgically treated trauma patients with tibial plateau fractures, who underwent rehabilitation according to PWB or RWB between 2005 and 2015. Data such as demographics, patient-reported quality of life and pain, and patient outcome were collected.

**Results:**

This cohort study included 91 patients with a tibial plateau fracture (31 and 60 patients in the PWB and RWB groups respectively). No significant between-group differences in either age or gender were found. However, a significant difference in fracture type was found between groups, (*p* = 0.04). No significant differences were found in either patient-reported SF-12 or VAS scores between the PWB group and RWB group. Time to full weight bearing was significantly shorter in the PWB than in the RWB group, i.e., 14.7 versus 20.7 weeks, (*p* = 0.02). No significant differences were found regarding postoperative complications between the PWB and the RWB groups, i.e., 6.5% versus 10.0%, respectively.

**Conclusion:**

PWB after surgically treated tibial plateau fractures is safe and is related to a significantly reduced time to full weight bearing with no significant differences in patient-reported quality of life and pain or complication rates.

## Introduction

The incidence of patients with tibial plateau fractures is approximately 13.3 per 100,000 [1]. Protocols for postoperative management of tibial plateau fractures were formulated about 60 years ago and suggest non- or partial weight bearing [2]. A survey about the adherence of current protocols showed that almost 90% of the surgeons do not follow these protocols standardly regarding the weight bearing aftercare for tibial plateau fractures [3]. In addition, patient’s compliance to a non- or partial weight bearing regimen is found to be poor and highly depending on the age of the patient [4, 5]. Elderly patients seem to be unable to maintain weight-bearing restrictions [6]. Thus, patients are likely to start weight bearing in an earlier phase than prescribed in current protocols.

The postoperative management of these surgically treated tibial plateau fractures in trauma patients is also very important regarding the functional outcome. The average overall postoperative complication rate in tibial plateau fractures, combining implant failures, secondary dislocation, non-union and infections into a composite end-point, is around 4–27% according to literature [7–14].

The standard aftercare treatment in surgically treated trauma patients with fractures of the tibial plateau features is non- or partial-weight bearing [15]. According to the Arbeitsgemeinschaft für Osteosynthesefragen (AO) principles of fracture management, postoperative management of tibial plateau fractures generally consists of toe-touch weight bearing for 6–8 weeks. As to fractures caused by extremely high-energy impact, these patients may need to adhere to toe-touch weight bearing regimen for 10–12 weeks [2]. There is currently no consensus among surgeons worldwide with regard to early weight bearing (i.e., permissive weight bearing) versus restricted weight bearing in surgically treated trauma patients with fractures of the tibial plateau [16].

Biomechanical and animal studies indicate that early weight bearing is beneficial [17–19], but high-quality clinical studies comparing permissive weight bearing (PWB) versus restricted weight bearing (RWB) after surgically treated tibial plateau fractures are scarce.

The aim of the present study was to inventory potential differences in quality of life and pain, and number of complications in patients with surgically treated tibial plateau fractures who followed a permissive weight bearing regime, relative to those that followed a restricted weight bearing regime.

## Patients and methods

This retrospective cohort study included surgically treated trauma patients with tibial plateau fractures at Maastricht University Medical Center+, the Netherlands, who underwent aftercare according the PWB or a RWB protocol between 2005 and 2015. In the PWB group, the patients were discharged to a rehabilitation center, where they were treated according the PWB protocol. Since 2003 PWB was gradually implemented and became standard care in our rehabilitation center from 2005. The fracture aftercare process starts by assessing the patient’s profile. Next, the generic and patient-specific treatment goals are identified, which, when combined, lead to the aftercare treatment aims. These aftercare treatment aims are then contrasted to the patient’s profile descriptors, which, together with potential predictors of surgically treated fracture aftercare outcome, may give insight into (a) the feasibility of the aftercare treatment aims; (b) the estimated time frame in which the aftercare treatment aims may be reached; and (c) the intensity/dosage/weight bearing needed to achieve the aftercare treatment aims. The increase in weight bearing is not based on a fixed percentage per week: weight bearing is gradually increased, based on the patient’s clinical presentation and with special attention to the quality of gait. Other key elements include body awareness and safe patient handling and moving algorithms, which are also considered to be key factors for successful treatment. The program involves multidisciplinary cooperation with surgeons, rehabilitation physicians and physical therapists, which is considered paramount to safely use the PWB protocol.

The patients included in the protocol suffered from two or more fractures (upper and lower extremity fractures), and therefore, needed more aftercare. The patients in the RWB group were discharged to their own home. They received passive exercise to maintain the muscles and the knee joint supported by a physical therapist, as prescribed by the surgeon.

All data in the study were collected from the electronic medical records by one researcher. Demographics of patients included age, gender and the presence of other fractures at the same time.

Primary outcome measures included the patient-reported questionnaire after at least 1-year follow-up; (1) Quality of life measured with the Short Form 12 (SF-12) [20]. The SF-12 consists of 12 items that assess eight dimensions of health: physical functioning, role-physical, bodily pain, general health, vitality, social functioning, role-emotional and mental health. The SF-12 measures various aspects of physical and mental health from which physical and mental summary scores can be calculated. (2) The intensity of pain measured with the VAS scale, (0 is no pain and 10 is worst pain) [21].

Time from surgery till full weight bearing and the total number and type of postoperative complications were collected from the electronic medical records. A postoperative complication was defined as a composite end-point comprising any complication, related to the fracture, that occurred during the aftercare regimen, these were recorded as either present or not present, along with the type of complications.

The medical ethics committee of Zuyderland Medical Center, Heerlen, the Netherlands approved this study and informed consent was given by all patients.

## Statistical analysis

Statistical analysis was performed with IBM SPSS Statistics, Version 23.0, Armonk, New York. Descriptive statistics were used to describe the demographic data and baseline characteristics of the entire population. Independent samples *t* tests were used for normally distributed continuous data and Chi-squared tests for categorical variables. Results are presented as either mean ± standard deviation (SD) or as frequencies and percentages. In case of non-parametric data the median with the interquartile range (IQR) are described. Binary logistic regression was performed to assess independent predictors of late full weight bearing (> 12 weeks) throughout both PWB and RWB groups. The level of statistical significance was set at *α* = 0.05.

## Results

### Baseline characteristics

This cohort study included 91 patients, 31 of whom were in the PWB group and 60 in the RWB group. Characteristics of patients in the PWB group and RWB group are presented in Table 1. Patients in the PWB group were significantly more likely to have a more complex fracture type [Schatzker fracture type (IV–VI) [22]] (*p* = 0.04) and more concomitant fractures than those in the RWB group (*p* < 0.01). No differences in age or gender were found between the two groups. Furthermore, no differences were found in surgical procedures between the two groups.

**Table 1:** Baseline characteristics of the PWB and RWB groups

	PWB (*N* = 31)	RWB (*N* = 60)	Total (*N* = 91)	*p*
Female	12 (38.7%)	27 (45.0%)	39 (42.9%)	0.66
Mean age (SD), years	50.4 (12.6)	50.9 (12.4)	50.8 (12.4)	0.86
≥ 2 fractures	26 (83.9%)	5 (8.3%)	31 (34.1%)	< 0.01
Schatzker types				
Type I–III	7 (22.6%)	27 (45.0%)	34 (37.4%)	0.04
Type IV–VI	24 (77.4%)	33 (55.0%)	57 (62.6%)	

*SD* standard deviation

### Patient-reported quality of life and pain

The overall response rate of the patient-reported questionnaire SF-12 and VAS scale was 72.5% (i.e., 66/91). No significant difference was found in response rate between the PWB group (80.6%) and RWB (68.3%) group (*p* = 0.32). The time between surgery and the moment at which the questionnaires administered was significantly higher in the RWB group than in the PWB group: 7.6 (3.2) years versus 4.6 (2.4) years (*p* < 0.01). No significant between-group differences were found in either quality of life measured with the SF-12 or the pain measured with the VAS scale (Table 2).

**Table 2:** Functional outcome measurements in the PWB and RWB groups

	PWB (*N* = 25)	RWB (*N* = 41)	Total (*N* = 66)	*p*
Mean SF-12 (quality of life) (SD)	58.0 (20.7)	68.8 (23.1)	64.7 (22.7)	0.06
Mean VAS scale (pain) (SD)	3.6 (2.2)	2.8 (2.7)	3.1 (2.5)	0.24

*SD* standard deviation

From the total population 38.5% of the patients (*N* = 35) reached full weight bearing within 12 weeks. The number of patients who reached full weight bearing within 12 weeks was significantly higher in the PWB group than in the RWB group: 58.1% versus 28.3% (*p* < 0.01). Time from surgery to ascertainment of full weight bearing was significantly shorter in the PWB group than in the RWB group: 14.7 (11.6) weeks versus 20.7 (11.5) weeks (*p* = 0.02) (Table 3). Binary logistic regression analysis revealed that, irrespective of PWB or RWB, Schatzker type and multiple fractures (*p* < 0.05) were independent predictors of late full weight bearing (> 12 weeks). No significant differences were found in time from surgery to full weight bearing between the specific fracture types (Schatzker type I–III versus Schatzker type IV–VI) (*p* = 0.10) in the PWB group (Table 4).

**Table 3:** Time to full weight bearing in the PWB and RWB groups

	PWB (*N* = 31)	RWB (*N* = 60)	Total (*N* = 91)	*p*
Within 12 weeks	18 (58.1%)	17 (28.3%)	35 (38.5%)	< 0.01
Mean time to full weight bearing (SD), in weeks	14.7 (11.6)	20.7 (11.5)	18.6 (11.9)	0.02

*SD* standard deviation

**Table 4:** Time to full weight bearing for specific fracture types in the PWB group

	Schatzker Type I–III (*N* = 7)	Schatzker Type IV–VI (*N* = 24)	Total Type I–VI (*N* = 31)	*p*
Within 12 weeks	6 (85.7%)	12 (50.0%)	18 (58.1%)	0.10
Mean time to full weight bearing (SD), in weeks	8.3 (5.1)	16.5 (12.4)	14.7 (11.6)	0.10

*SD* standard deviation

### Patient outcome

No significant differences were found in the incidence of postoperative complications between the PWB group and the RWB group, values of which were 6.5% (*N* = 2) versus 10.0% (*N* = 6), respectively. In the PWB group. The complications in the PWB group consisted of *N* = 1 non-union and *N* = 1 superficial wound infection. It should be noted, however, that both patients started full weight bearing after the postoperative complication. The complications in the RWB group consisted of *N* = 3 non-unions, *N* = 2 superficial wound infections and *N* = 1 deep infection. Furthermore, no significant differences between the PWB group and RWB group were found regarding either the postoperative removal of osteosynthesis material or the number of total knee prostheses (Table 5).

**Table 5:** Patient outcome measurements in the PWB and RWB groups

	PWB (*N* = 31)	RWB (*N* = 60)	Total (*N* = 91)	*p*
Total postoperative complications	2 (6.5%)	6 (10.0%)	8 (8.8%)	0.58
Postoperative ROSM	7 (22.6%)	24 (40.0%)	31 (34.1%)	0.10
Postoperative TKP	5 (16.1%)	5 (8.3%)	10 (11.0%)	0.27

*SD* standard deviation, *ROSM* removal osteosynthesis material, *TKP* total knee prosthesis

## Discussion

This retrospective cohort study found that the use of a PWB protocol for patients with a surgically treated tibial plateau fracture was associated with reduced time to full weight bearing, while similar quality of life, pain and postoperative complication rates were found, compared to RWB. Furthermore, no significant differences were found in rates of postoperative removal of osteosynthesis material or the need for total knee prostheses after tibial plateau fractures.

In our study 28.3% of patients in the RWB were already bearing full weight within 12 weeks, highlighting the contrast to the standard protocol of 12 weeks non-weight bearing. The patients in the PWB group were already bearing full weight 6 weeks earlier than the RWB group. In addition, earlier studies reported that one-third of the patients do not (fully) comply to a non- or limited weight bearing regimen [4, 5]. A number of studies found patients to exceed the prescribed amount of partial weight bearing even when self-reported compliance was high [23]. Despite the willingness to comply, patients often do not follow the restrictions in weight bearing and advance their weight bearing as fracture healing progresses.

During normal daily activities the knee joint experiences forces between 220 and 350% of a person’s body weight. As even a 3-mm step-off in the tibial plateau can increase the cartilage contact stresses by 75%, concerns are raised that loss of reduction could lead to worse patient outcomes, even in case of non-weight bearing [24]. On the other hand, it is often stated that early weight bearing does not pose an undue risk of complications or worse patient outcomes compared to a non-weight bearing protocol, as reported in a recent randomized controlled trial dealing with fractures of the ankle joint [25]. These two statements are contradictory and require further elaboration. Our study adds evidence in favor of regimens with earlier than standard postoperative weight bearing protocols and shows that there is no significant difference in quality of life, pain or complications compared to RWB.

One of the key objections against early weight bearing is the possibility of fracture displacement [26]. In one radiostereometric study at 1 year after early weight bearing of fractures of the tibia plateau, the mean craniocaudal migration of the fracture fragments was − 0.34 mm (− 1.64 to 1.51) [27]. This case series has shown that, in the Schatzker type II fractures investigated, internal fixation with subchondral screws and a buttress plate provided adequate stability to allow immediate postoperative partial weight-bearing, without harmful consequences.

Longer term outcomes have as well been described in the literature, with more favorable results for PWB. In a prospective, multicenter randomized trial involving bicondylar tibial plateau fractures, a group of 43 patients underwent fixation with external ring fixation and were permitted to bear full weight, while a group of 40 patients underwent open reduction and internal fixation with restricted weight bearing [8]. At a minimum 2-year follow-up, there was no difference in reoperations, articular incongruity, or development of radiographic signs of osteoarthritis between the two groups. In line with this study, our study found that there were no significant differences in pain or reoperations (removal of osteosynthesis material or implants of total knee prostheses). Interestingly, removal of osteosynthesis material in the PWB group was lower than in RWB group, i.e., 22.6% versus 40.0%, respectively.

According to recent literature, a composite postoperative complication rate of up to 27% has been reported in tibial plateau fractures [7–14]. Comparing our complication data with data published in recent literature, we found decreased rates of postoperative complication in tibial plateau fractures treated by means of a PWB protocol, despite the fact that more severe fractures were found in our PWB population. The latter could be an explanation for the fact that the other 41.9% of the PWB population did not reach full weight bearing within 12 weeks, which might be due to a high-comorbidity rate of our PWB population. Nevertheless, the average time to full weight bearing was significantly lower in the PWB group than the RWB group.

Over and under-loading may lead to prolonged and complicated recovery. A certain minimum level of loading is required to elicit micro-movements between adjacent bony fracture components, stimulating biological processes that enhance fracture consolidation and minimizing effects of immobilization [28, 29]. To optimize recovery with the lowest number of complications we want to set out a treatment that is near to the upper boundary of the therapeutic bandwidth regarding weight bearing, yet safe enough to avoid complications regarding overloading.

Our study, the first study comparing PWB with RWB, adds evidence in support of the use of PWB in patients with surgically treated tibial plateau fractures. However, limitations in our study include the retrospective nature of the study and, due to this retrospection, not taking into account surgeon-oriented functional outcome scores (e.g., knee function) or generic patient satisfaction scores. Furthermore, no radiological controls have been done to investigate the alignment of the fractures. Another limitation of the study is the lack of monitoring patient compliance. To mitigate these limitations, we have started a prospective cohort study in patients with fractures of the lower extremities [30].

## Conclusion

This retrospective cohort study shows that permissive weight bearing after surgically treated tibial plateau fractures is safe and is related to a significant reduced time to full weight bearing with no significant differences in patient-reported quality of life and pain or complication rates.
